# Analyzing the Relationship Between Job Stress to Mental Health, Personality Type And Stressful Life Events of the Nurses Occupied in Tehran 115 Emergency

**DOI:** 10.5812/ircmj.1917

**Published:** 2013-03-05

**Authors:** Hadi Tehrani, Tayebeh Rakhshani, Davood Shojaee Zadeh, Seyed Mostafa Hosseini, Samane Bagheriyan

**Affiliations:** 1 Teheran University of Medical Science, Tehran, IR Iran; 2 Kerman University of Medical Sciences, Kerman, IR Iran

**Keywords:** Mental Health, Type A Personality, Life Change Events, Emergencies

Dear Editor,

Nursing are known as a profession that is constantly exposed to various stresses. Stress job in this profession could lead to disturbances that seriously put at risk the mental health of nurses. Pressure caused by stressors in the job has the undesirable effect on the individual and the organization. Increased accidents and injuries, poor staff morale, low motivation, job satisfaction and Fatigue early are natural consequence lack of the elimination of workplace stressors (1). Although nurses have been trained to attention to care and quality of life of patients But they rarely think of their individual needs (2). Occupational Safety and Health Institute has been investigated the relationship between mental illness and job stress , reported that Among the 130 job studied, Nursing has been allocated ranked 17th in the acceptance rate to mental health problems (3). National immunity Association of America has also reported that amongst 40th most stressful professions, nursing job is with high prevalence of disease-related stress (4). Therefore we conducted a cross-sectional study over 200 randomly selected nurses which working in Tehran Emergency Medical Services (Also named 115 Tehran Emergency units) to investigate the relationship between psychological stress and personality type and life events in them. A general Health questionnaire and a special questionnaire were used in order to evaluate their mental health and possible contributing factors including job stress personality type and daily life events respectively. Results of the study show that 115 emergency nurses in Tehran are facing with high job stress. Mental health reduce, When higher levels of job stress and exposure to stressful life events increase in this group, The findings indicate that increasing in the stressful life events cause to job stress is more and People with type A personality are less compatible with stress and are prone to get mental disorder. Overall, the results of this study are consistent with other studies in the field of occupational stress such as Gershon E (5), Kirkcaldy D (6) and Neylon TC (7). However, it is important to be noted that these studies have been carried out on populations with different characteristics and various stressful life. This study showed that there is a significant relationship between job stress with mental health and exposure time of life events with personality type. In general, mental health nurses in the emergency 115 is at risk more than other segments of society is at risk, the main reasons can be noted such as; stressful nature of the profession, Working pressure, dealing with unexpected situations, shifts, extra responsibility, Excessive demands from the patient and his family, Lack of recreational facilities, Visionary with the reality of death, Organizational factors and individual factors . Prevalence of impaired health nurses reported different numbers in other parts of the world for example Rout showed The major sources of stress isolated by the ward nurses related to: time pressure, administrative responsibility, having too much to do, component not under their control, interruptions, keeping up with National Health Service (NHS) changes, and lack of sources (8) Farrell have reported The prevalence of mental health disorder among nurses 34 percent and Fagin stated 41 percent in nurses, (9, 10) Researches conducted in other countries stated The prevalence of mental health disorder in nurses is higher than the other social groups. For example Yang reported the prevalence of mental health disorder in the nursing population 48/8 % against the general population of 33/3% (11). In this study, there was no significant difference between job stress and mental health status with Professional Background. It was noticed that the nursing staff with a bachelor's degree in nursing and operating room technicians were better in job stress and mental health perhaps attributed to self-confidence and pays more than them. The survey also showed that between marital status and mental health and job stress are significant relations. The survey also showed that between marital status and mental health and job stress are significant relations so that the single people had better mental health and job stress which can be caused by having generally more family complications and responsibilities, which generally leads to more stress as compared to those who are single (12). However, the necessity of maintaining social, occupational and educational functioning is mental health in community. In recent decades the main issues of organization's management has allocated to mental health and its impact on an organization. Stress in organizations such as aphthous disrupts their activity and efforts. So much capital is lost due to lack of physical - mental health staff, reducing their efficiency and absence from work. The results of this study could be considered as a data source to be used for planning in mental health programs.
